# The use of local therapy in preventing urethral strictures: A systematic review

**DOI:** 10.1371/journal.pone.0258256

**Published:** 2021-10-06

**Authors:** Marleen E. Jacobs, Vincent F. de Kemp, Maarten Albersen, Laetitia M. O. de Kort, Petra de Graaf

**Affiliations:** 1 Department of Urology, University Medical Center Utrecht, Utrecht, The Netherlands; 2 Department of Urology, University Hospital Leuven, Leuven, Belgium; 3 Regenerative Medicine Utrecht, Utrecht, The Netherlands; Mayo Clinic Rochester: Mayo Clinic Minnesota, UNITED STATES

## Abstract

**Background:**

Urethral stricture disease is a common problem amongst men in Western countries often leading to a decreased quality of life. Current endoscopic treatment procedure shows an unsatisfying stricture recurrence rate which could be improved by addition of local therapies.

**Objectives:**

To provide an overview of both preclinical and clinical studies in order to investigate current level of evidence on the addition of local therapy to improve urethral stricture recurrence rates after endoscopic procedures.

**Methods:**

We performed a literature search in December 2020 and August 2021 using Cochrane, Embase, PubMed, Scopus and Web of Science and identified articles through combinations of search terms for ‘urethral stricture disease’, ‘stricture formation’ and ‘local interventions’. We used the SYRCLE, RoB-2 and ROBINS-I tools to assess risk of bias across included studies. We did not perform a meta-analysis due to methodological differences between studies.

**Results:**

We included 32 articles in the qualitative analysis, 20 of which were preclinical studies and 12 clinical studies. Regarding preclinical articles using an animal model, nearly all interventions showed to have a positive effect on either urethral fibrosis, urethral stricture formation and/or fibrotic protein expression levels. Here, immunosuppressants and chemotherapeutics seemed most promising for possible clinical purposes. Regarding clinical studies, mitomycin-C and hyaluronic acid and carboxymethylcellulose showed positive effects on urethral stricture recurrence rates with low to intermediate risk of bias across studies. However, the positive clinical effects of mitomycin-C and steroids seemed to decrease in studies with a longer follow-up time.

**Conclusion:**

Although local adjuvant use of mitomycin-C or hyaluronic acid and carboxymethylcellulose may carry clinical potential to improve urethral structure recurrence rates after endoscopic procedures, we believe that a large, well-designed RCT with a yearlong follow-up time is necessary to identify the true clinical value.

## Introduction

Urethral strictures (US) are sections of narrowed urethral lumen caused by excessive scar formation of the urethral wall [1]. Current prevalence of male US disease is estimated between 0.6 and 1.4% [2,3]. In Western countries, aetiology of US disease is mostly idiopathic followed by iatrogenic; developing countries show higher prevalence of traumatic US and infectious strictures after (bacterial) urethritis [1,4]. Presence of US can reduce quality of life by causing obstructive voiding symptoms and possibly damage to the urinary tract [1].

Current US disease treatment options comprise both endoscopic and open surgical procedures with varying success rates. The endoscopic direct visual internal urethrotomy (DVIU) includes incision of the US to enlarge the urethral lumen and is predominantly used for the initial treatment of short (<1–1,5cm) bulbar strictures [4]. Although endoscopic interventions are most frequently used, results are often disappointing with high long-term structure recurrence rates of 60 to 80 percent [5]. Longer (>2cm), non-bulbar or recurrent short anterior strictures unsuccessfully treated with DVIU are often treated with open reconstructive procedures with improved success rates but at the cost of being more invasive [4,6]. Additionally, open procedures require a higher level of surgical expertise compared to endoscopic procedures and can be less suitable for a frail subset of patients with high age of comorbidities in the population with US disease [4]. Therefore, optimalisation of current endoscopic procedures is necessary.

Although multiple experimental studies and clinical trials have investigated the effectiveness of additional local therapies to improve endoscopic success rates, a recent overview assessing possible efficacy of different local interventions is lacking [7,8]. This systematic review aims to provide a complete overview of both preclinical and clinical studies in order to investigate current level of evidence on the addition of local therapy to improve US recurrence rates after endoscopic procedures.

## Methods

### Literature search

We performed a systematic literature search in December 2020 and August 2021 using Cochrane, Embase, PubMed, Scopus and Web of Science. We identified articles through combinations of MeSH terms, Emtree terms and related terms for ‘urethral stricture disease’, ‘stricture formation’ and ‘local interventions’. S1 File provides our elaborate search strategy. Type of study subjects (animal, human) and publication year formed no restriction for the search. Regarding the Web of Science database, we restricted the search to specific article formats. We supplemented the literature search results with a few additional cross references.

### Study selection and risk of bias analysis

We imported all articles retrieved from both literature searches using Rayyan QRCI software and removed duplicates. Two authors independently performed title and abstract screening (MJ, PdG); three authors independently performed full text screen using pre-defined eligibility criteria (MJ, VdK, PdG) (S2 File). We resolved any mismatches in authors’ screening results through discussion. To assess methodological quality of included studies, we performed a risk of bias (RoB) analysis using the SYRCLE’s RoB tool for preclinical studies and the Cochrane RoB-2 tool and ROBIN-I for clinical studies [9–11].

### Data extraction

After study selection, we retrieved relevant outcomes from all articles. Regarding preclinical studies, we defined morphological changes in histopathological analysis related to US formation as the primary outcome measure. We defined both formation of US as well as changes in US-related protein expression as secondary outcomes. Regarding clinical studies, we defined the primary outcome as the recurrence of US. We did not combine outcomes in a meta-analysis because of the elaborate methodological differences between included studies.

## Results

### Search results

**Fig 1:**
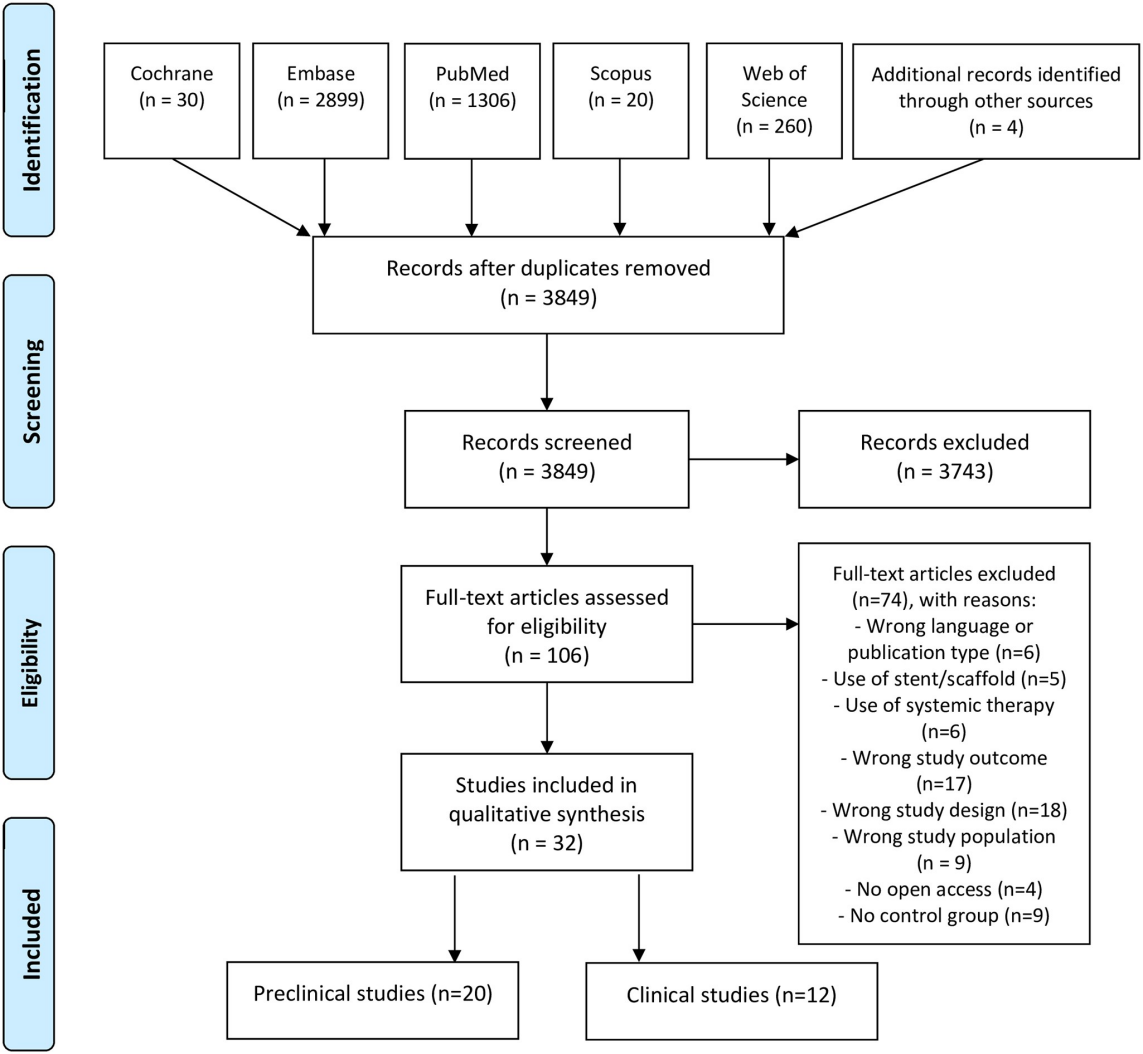
Study selection process [12].

Our literature search yielded a total of 4521 articles of which 3849 remained after removal of exact duplicates and addition of cross references. Title and abstract screening brought up a total of 106 studies included in the full text screen of which we selected 32 articles for inclusion in the qualitative analysis. From these 32 included articles, 20 evaluated the use of local therapy in an animal model while 12 included human studies. Fig 1 shows the study selection process [12].

#### Preclinical studies

Table 1 displays study outcomes from 20 included preclinical studies. All studies used rats or rabbits as an animal model with induction of US through either electrocoagulation, mechanical induction or use of a pro-fibrotic agent. Regarding the type of local therapy, studies used either an extracellular matrix (ECM) modulator (n = 5), chemotherapeutics (n = 4), cell-derived material (n = 4), immunosuppressants (n = 2) or any other local drug (n = 6) to modulate histological outcomes related to US formation.

**Table 1:** Overview of preclinical study outcomes.

Study	Subjects (experimental/control)*	Method of US induction	Topical intervention, specified	FU time (weeks)	Relevant outcome(s)**
*ECM modulators*					
Guzmán-Esquival *2011* [13]	17 rabbits (6/11)	EC	857,1 ng MMP, once at t = 14 days	3,5	16% decrease in collagen content in MMP-group (1) Significantly larger urethral area in the MMP-group, compared to the PBS-instilled rabbits (2)
Krane *2011* [14]	12 rats (6/6)	EC	0.5 mg/mL halofuginone, catheter coated	2	Absence of urethral collagen deposition in halofuginone groups (1)
Nagler *2000* [16]	18 rats (12/6)	EC	0.03% halofuginone, once a day for 7 days	3	Decrease in collagen protein content compared to the positive control group (1) Decrease in US formation in group treated with halofuginone compared to the positive control group (2) Decrease in collagen protein expression compared to the positive control group (3)
Sangkum *2015* [15]	30 rats (16/14)	TGF-β1	Either 0.05mg (L) or 0.1 mg (H) CCH, daily for 14 days	4	Decrease in submucosal fibrosis in the CCH-H group (1) Decrease in collagen protein expression in CCH-L and CCH-H groups (3)
Shinchi *2019* [17]	19 rabbits (7/12)	EC	IGF-1 impregnated collagen, catheter coated	2	Decrease of histological urethral damage in IGF-1 group (1) Decrease of US formation in IGF-1 group (2)
*Chemotherapeutics*					
Ayyildiz *2004* [18]	35 rats (30/5)	Mechanically	Either 2mg/L MMC or 10mg/L MMC, once	2	Decrease in chronic inflammation and urethral fibrosis in both MMC groups (1)
Chang *2015* [19]	20 rats (5/15)	Mechanically	3mg/L MMC, daily for 14 days	2	Decrease in urothelial proliferation and wound healing activity in the MMC group (1)
Fu *2014* [20]	36 rabbits (29/7)	EC	Either 0.01mg or 0.1mg docetaxel, daily for 28 days	4	Decrease in fibrotic content in both low- and high-dose groups (1) Decrease in US formation in both low-dose (37% vs. 100%) and high-dose (0% vs. 100%) docetaxel groups (2)
Kurt *2017* [21]	16 rabbits*** (8/8)	Mechanically	0.5mg/mL MMC, once	4	Decrease in fibrosis and collagen bundle irregularity in the MMC group (1)
*Cell-derived material*					
Castiglione *2016* [22]	36 rats (12/24)	TGF-β1	10^6^ hADSCs, once	4	Decrease in collagen and elastin levels in the hADSC group (1) Decrease in US formation in the hADSC group (2)
Sangkum *2016* [25]	9 rats (6/3)	Mechanically	2*10^5^ ADSCs, once	2	Decrease in submucosal fibrosis in the ADSC group (1) Decrease collagen type I/III expression in the ADSC group (3)
Nikolavsky *2016* [23]	18 rats (6/12)	TGF-β1	Liquid BMG, once	8, 16, 24	Significant improvement or complete resolution of US in BMG treated group (100% vs. 33%) (2)
Shi et al *2020* [24]	45 rats (15/30)	TGF-β1 injection and mechanically	BMSC derived exosomes, once	4	Decrease of urethral fibrosis in the BMSC group (1) Decrease in US formation in the BMSC group (2) Decrease in gene expression in fibrosis-related markers in the BMSC group (3)
*Immunosuppressants*					
Chong *2011* [26]	37 rabbits (24/13)	EC	Either 0.1mg or 1.0 mg rapamycin, daily for 28 days	4	Decrease in fibrosis and collagen deposition in both low- and high dose groups (1) Decrease in US formation in both the low-dose (67% vs. 100%) and the high-dose (17% vs. 100%) groups (2)
Kurt *2017* [21]	16 rabbits*** (8/8)	Mechanically	40mg triamcinolone, once	4	Decrease in fibrosis and collagen bundle irregularity in the triamcinolone group (2)
*Other*					
Ayyildiz *2007* [27]	40 rats (24/16)	Mechanically	1mL 10% honey solution, daily for either 7 or 21 days	3	Improvement in urethral coverage, healing of spongiosa tissue and total healing after 21 days treatment (1) Improvement in urethral narrowing after 21 days treatment (2)
Dündar *2002* [32]	14 rats (7/7)	Mechanically	10mg/kg diltiazem, daily for 5 days	3	No difference in connective tissue thickness, epithelial lining and degree of inflammation between groups (1)
Kilinc *2019* [29]	30 rats (20/10)	Insulin injector	Either 25 IU/kg EPO twice a day or 25IU/kg EPO daily + 0.9% saline daily for 14 days	2	Decrease in spongiofibrosis and inflammation in both EPO groups (1)
Sahinkanat *2009* [30]	30 rats (20/10)	Mechanically	BTX-A injection, once	3	Decrease in urethral fibrosis in the BTX-A group (1)
Yardimci *2015* [28]	27 rats (18/9)	Mechanically	Either 500mg/kg dexpanthenol twice a day or 500mg/kg dexpanthenol daily + 0.9% saline daily for 14 days, catheter coated	2	Decrease in urethral fibrosis in the high-dose dexpanthenol and decrease in inflammation in both the low- and high-dose dexpanthenol groups (1)
Yildizhan *2020* [31]	40 rats (30/10)	Mechanically	1 mL of either sodium hyaluronate 1.8% or sodium hyaluronate 3%, daily for 5 days	3	Decrease in tissue thickness in both hyaluronate groups compared to the positive control group (1)

*Abbreviations*: ADSC: Adipose derived stem cells, BMG: Buccal mucosal graft, BMSC: Bone marrow derived stem cells, BTX-A: Botulinum toxin-A, CCH: Collagenase clostridium histolyticum, DNA: Deoxyribonucleic acid, EC: Electrocoagulation, EMC: Extracellular matrix, EPO: Erythropoietin, FU: Follow-up, IGF-1: Insulin-like growth factor 1, MMC: Mitomycin-C, MMP: Metalloproteinase-I, PBS: Phosphate buffered saline, TGF-β1: Transforming growth factor beta 1, US: Urethral stricture.

* Only subjects that completed the follow-up time were included in this section.

** We defined morphological changes in histopathological analysis as primary outcome (1); US formation (2) and changes in US-related protein expression (3) as secondary outcomes.

*** In total, 24 rats were included in this study. Both experimental groups (MMC and triamcinolone, n = 8) were compared to the same control group (n = 8).

### ECM modulators

Through application of either a single dose, daily administration or delivery through catheter impregnation, ECM modulators showed to decrease urethral fibrosis or collagen content in four studies [13–16] and prevent the formation of US in two different studies (2–4 weeks follow-up (FU)) [16,17]. Two studies performed additional analyses on collagen protein expression showing a decrease in urethral expression levels after local ECM modulation [15,16]. Furthermore, the use of high dose collagenase clostridium histolyticum increased histopathological effects on submucosal fibrosis content compared to a lower dosage [15].

### Chemotherapeutics

Both single application and daily instillation of mitomycin-C (MMC) in varying dosages caused a decrease in histopathological fibrosis, inflammation and urothelial proliferation after a 2–4 week FU period [18–21]. In addition to a decrease in fibrotic content, daily application of docetaxel caused a decrease in US formation with larger effect in high-dosed animals (FU 4 weeks) [20].

### Cell-derived material

Single application of adipose derived stem cells caused a decrease in submucosal fibrosis and collagen and elastin content in two studies with a 2–4 weeks FU period [22,23]. One of these studies also showed a decrease in US formation [22]; the other a decrease in collagen type I/III expression [23]. Single use of bone marrow derived stem cell exosomes caused a decrease in fibrosis, US formation and fibrosis-related gene expression (4 weeks FU) [24]. Lastly, with a FU period up to 24 weeks, single application of liquid buccal mucosal graft also caused an improvement in US formation [25].

### Immunosuppressants

Either single or daily application of immunosuppressants caused a decrease in urethral fibrosis and reduced content of (irregular) collagen bundles in two studies (FU 4 weeks) [21,26]. In one of these studies, US formation also decreased with larger effects of high dosage rapamycin treatment compared to low dose treatment [26].

### Other

Daily application of a lubricating honey solution caused an improvement in urethral coverage, healing and narrowing after 21 days [27]. The use of dexpanthenol as catheter lubricant caused a decrease in urethral fibrosis in case of high dose treatment and a decrease in inflammation in both low and high dosage groups [28]. Daily application of erythropoietin (EPO) or a single injection with botulinum toxin-A in urethral mucosa caused decrease in urethral fibrosis, with no increasing effect in high dosage EPO animals [29,30]. Both low and high dose daily application of sodium hyaluronate (5 days total) decreased urethral tissue thickness after 21 days [31]. Lastly, daily use of a calcium antagonist, diltiazem, for 5 days did not result in any significant histopathological changes after 21 days (connective tissue thickness, epithelial lining and degree of inflammation) [32].

### RoB analysis

Using SYRCLE’s tool for animal studies, we performed a RoB on all 20 included studies (S3 File) [9]. Overall, we scored 4/20 studies as ‘low’, 9/20 as ‘intermediate’ and 7/20 as ‘high’ RoB. Comparing subsets of articles using the same kind of local intervention, studies on ECM modulators showed the highest overall RoB. Here, 3/5 studies had a high score because of inaccuracies during sequence generation, animal housing and/or inadequate blinding of outcome assessors resulting in risks on selection and performance bias. In the second study subset (chemotherapeutics) no studies showed a high overall RoB while 3/4 studies scored an intermediate RoB due to lack of information on animal allocation, housing and outcome assessment. Here, RoB in other fields was either low or intermediate. Three studies on cell-derived materials showed either a low or intermediate overall RoB, while one study using adipose derived stem cells scored an overall high RoB mainly as a result of inadequate animal allocation and lack of blinding during outcome assessment. Additionally, one study from the “other” subset using botulinum also scored a high overall RoB based on the same inaccuracies. Two studies from the “other” subset also scored a high overall RoB due to lack of inclusion of animal baseline characteristics. The other four studies from the immunosuppressant and other article subsets scored varying overall RoB scores due to alternating risks on selection, performance and detection bias.

#### Clinical studies

Table 2 displays study outcomes from 12 included clinical studies with either a controlled or randomized controlled (RCT) study design. All local interventions were used during or after endoscopic interventions for the treatment of US disease. Regarding the type of local therapy, studies used either an ECM modulator (n = 1), chemotherapeutics (n = 3), immunosuppressants (n = 7) or any other form of local intervention (n = 2) to modulate US development or recurrence.

**Table 2:** Overview of clinical study outcomes.

Study	Study type	Subjects (experimental/control)*	Topical intervention, specified	FU time (months)	Relevant outcome(s)**,***	P-value
*ECM modulators*						
Ergün *2015* [33]	RCT	60 patients with US, undergoing IU (30/30)****	Contractubex ointment CIC, once a week for 6 weeks (duration 5 minutes)for 6 weeks	24	Decrease in US recurrence rate (23.3% vs. 33.3%) ^†^	>0.05
*Chemotherapeutics*						
Ali *2015* [35]	RCT	151 patients with anterior US, undergoing IU (78/73)	0.1% MMC, submucosal injection, directly after IU	18	Decrease in US recurrence rate (14.1% vs. 36.9%) †, ‡, §	0.002
Mazdak *2007* [36]	RCT	40 patients with anterior US, undergoing IU (20/20)	0.05mg/mL MMC, submucosal injection, directly after IU	6	Decrease in US recurrence rate (10% vs. 50%) †, §	0.006
Moradi *2016* [34]	RCT	40 patients with US, undergoing IU (20/20)	0.8mg/mL MMC, hydrogel installation, directly after IU	12	Decrease in US recurrence rate (10% vs. 50%) †, §	0.001
*Immunosuppressants*						
Ergün *2015* [33]	RCT	60 patients with US, undergoing IU (30/30)***	Triamcinolone ointment CIC, once a week for 6 weeks (duration 5 minutes)	24	Decrease in US recurrence rate (30% vs. 33.3%) ^†^	>0.05
Hosseini *2008* [42]	RCT	64 patients with US, undergoing IU (30/34)	1% triamcinolone ointment CIC, repeatedly following treatment regimen for 6 months (duration unknown) *****	12	Decrease in US recurrence rate (30.0% vs. 44.1%) †, §, ¶	0.24
Korhonen *1990* [37]	Controlled clinical trial	38 patients with US, undergoing IU (17/31)	40–120 mg methylprednisone, submucosal injection, directly after IU	12	Decrease in US recurrence rate (81% vs. 100%) ^¶^	NR
Mazdak *2010* [38]	RCT	45 patients with anterior US, undergoing IU (23/22)	40mg triamcinolone, submucosal injection, directly after IU	12	Decrease in US recurrence rate (50% vs. 21.7%) ^¶^	0.04
Regmi *2018* [41]	RCT	55 patients with US, undergoing IU (27/28)	1% triamcinolone ointment CIC, repeatedly following treatment regimen for 6 months (duration unknown)*****	12	Decrease in US recurrence rate (22.22% vs. 46.42%) †, §, ¶	0.04
Tavakkoli Tabassi *2011* [39]	RCT	70 patients with US, undergoing IU (34/36)	5mg triamcinolone, submucosal injection, directly after IU	6–24	Decrease in US recurrence rate (35.3% vs. 41.7%) ^¶^	0.584
Yildirim *2016* (4039)	Controlled clinical trial	83 patients with US, undergoing IU (34/36)	40–80 mg methylprednisone, directly after IU	18 (at least)	Decrease in US recurrence rate (5% vs. 28%) †, ‡	NR
*Other*						
Chung *2013* [43]	RCT	101 patients with US, undergoing IU (53/48)	5mg HA/CMC hydrogel installation, directly after IU	6	Decrease in US recurrence rate (22.9% vs. 9.4%) †, ‡, §	0.029
Shirazi *2007* [44]	Controlled clinical trial	56 patients with US, undergoing IU (37/19)	Either 0.1% or 0.5% captopril gel installation, repeatedly following treatment regimen for 6 weeks******	6–30	Decrease in US recurrence rate in both high- (35.3% vs. 52.6%) and low-dose (30% vs. 52.6%) captopril groups †, ‡, §	<0.05, <0.05

*Abbreviations*: CIC: Clean intermittent catheterization, ECM: Extracellular matrix, FU: Follow-up, HA: Hyaluronic acid, HA/CMC: Hyaluronic acid and carboxymethylcellulose, IU: Internal urethrotomy, MMC: Mitomycin-C, NR: Not reported, RCT: Randomized controlled trial, US: Urethral stricture.

* Only patients that completed the follow-up time were included in this section.

** Outcomes are displayed as experimental group vs. control group, unless specified otherwise.

*** In clinical studies, US recurrence was defined as recurrence of clinical symptoms (†), decrease in urethral flow rate measured by urethroflowmetry (‡), retrograde urethrography or urethrocystoscopy proven US recurrence (§) and/or the need for secondary procedure, such as repeated urethrotomy or urethroplasty (¶).

**** In total, 90 patients were included in this study. Both experimental groups (contractubex and triamcinolone, n = 30) were compared to the same control group (n = 30).

***** CIC treatment regimen was set as following: 1^st^ week daily, 2^nd^ week every other day, 3^rd^ week twice a week, 4^th^ week once a week, 2^nd^ month every two weeks and 3^rd^– 6^th^ month once a month.

****** Treatment regimen was set as following: 1^st^ week daily, 2nd– 3^rd^ week every other day, 4^th^– 6^th^ week twice a week.

### ECM modulators

As reported in one study including 60 patients, use of contractubex ointment for weekly clean intermittent catheterization (CIC) caused no decrease in US recurrence rates after FU period of 24 months [33]. Here, weekly CIC was performed for 6 weeks total (duration 5 minutes per session).

### Chemotherapeutics

Two studies including a total of 191 patients applied a single submucosal injection of MMC directly after DVIU; one study including 40 patients used a single gel installation directly after DVIU [34–36]. In all three studies this led to a significant decrease in US recurrence rate (FU period 6–18 months).

### Immunosuppressants

Four studies including a total of 236 patients assessed single application of immunosuppressant (triamcinolone or methylprednisone) submucosally injected directly after DVIU with a FU period of 12–24 months [37–40]. Here, only one out of four studies reported a significant decrease in US recurrence rate [38]. One other study showed no decrease in US recurrence rate after triamcinolone application [39]; the other two non-randomized controlled trials using methylprednisone did not report significance analyses [37,40]. Furthermore, three studies used triamcinolone ointment for CIC in a total of 179 patients with a FU period of 12–24 months [33,41,42]. Two studies reported specific 6 months CIC treatment regimens with unknown duration of a single catheterization; one study performed weekly CIC for 6 weeks with a duration of 5 minutes per intervention. Only one of these three studies reported a decrease in US recurrence rate after a six-month treatment regimen while the other two showed no decrease in US recurrence after treatment regimens of 6 weeks and 6 months, respectively [33,41,42].

### Other

Regarding other local interventions, one study including 101 patients assessed the effect of single application of hyaluronic acid and carboxymethylcellulose (HA/CMC) gel installation on recurrence of US after DVIU which led to significant reduction of US recurrence after 6 months FU [43]. Furthermore, repeated local administration of both low- and high doses captopril gel installation following a 6-week treatment regimen in a total of 56 patients caused a decrease in US recurrence rate after DVIU (FU period 6–30 months) [44].

### Risk of bias analysis

Using the RoB-2 tool for randomized controlled clinical trials and the ROBINS-I tool for controlled clinical trials, we scored all 12 clinical studies with a ‘low’ (n = 2), ‘intermediate’ (n = 8), ‘intermediate/high’ (n = 1) or ‘high’ (n = 1) RoB (S4 and S5 Tables in S4 File). Comparing subsets of articles using the same kind of local intervention, studies on immunosuppressants scored the highest overall RoB. Regarding one study on local use of an ECM modulator, overall RoB was intermediate because of inaccuracies in methods of outcome measurement. Regarding chemotherapeutics, three studies scored an intermediate overall RoB, mainly due to knowledge of intervention by either the patients or assessors, loss to follow-up and/or inaccuracies during outcome measurements. Except for one study scoring a high RoB due to inadequate experimental blinding, overall scores in RCTs using immunosuppressants were mainly intermediate as a result of deviations from intended interventions and inaccuracy in outcome measurements. In controlled clinical trials, immunosuppressant studies showed an intermediate RoB because of unclear methods of patient distribution across groups. Lastly, regarding other interventions, one study assessing HA/CMC as local therapy scored an overall low RoB while the study on captopril gel scored an intermediate to high risk due to missing outcome data.

## Discussion

To our knowledge, this is the first study that summarizes both preclinical and clinical research while including a broad spectrum of possible local therapies to improve the endoscopic treatment for US disease. Because of our elaborate search strategy we are confident that we have yielded all relevant literature on this topic. With inclusion of more than 850 patients from 12 different clinical studies, we provide a clear overview of currently available clinical evidence. As the majority of local therapies can be applied through single injection or installation during DVIU, these interventions are easy to implement in current treatment methods. Lastly, inclusion of 20 preclinical studies contributes to insight in clinical potential and working mechanism of a wide range of local treatments.

Although not every preclinical study reported the direct effect of local additional treatment on US formation, 19/20 studies showed positive results on urethral tissue thickness, urethral fibrosis and/or collagen deposition. Taking the outcomes from the RoB into account, the use of chemotherapeutics, in particular MMC, and immunosuppressants showed to have positive effects while carrying a low overall RoB across studies. Giving the cost effectivity of both treatments and the fact that studies reported little to no adverse events in treated animals, MMC and immunosuppressants may be most promising for clinical purposes. Acknowledgment of the positive effects of MMC in preclinical trials is in line with previous research [7]. Furthermore, the use of dexpanthenol and bone marrow derived stem cell exosomes showed positive results in two studies with an overall low RoB. Here, we believe that dexpanthenol may carry beneficial dose-dependent effects for clinical purposes while the clinical use of exosomes is limited through its relatively low cost effectivity.

Regarding clinical trials, we believe that the use of MMC and HA/CMC may carry potential as an additional treatment in endoscopic procedures to decrease US recurrence rate. All studies focusing on MMC or HA/CMC reported positive results while carrying a low to intermediate overall RoB. In our opinion, both therapies seem cost effective with little risks on adverse treatment events. This positive clinical effect of MMC was also acknowledged in a similar review specifically summarizing clinical studies on this subject [45]. Furthermore, the use of HA/CMC has also shown positive results in prevention of US *development* after endoscopic prostate surgery, strengthening our evidence [46]. In line with previous research, we found varying effects of local steroids on US recurrence rate in studies with an overall intermediate to high RoB [8]. Interestingly, clinical effects seemed to decrease in trials with longer follow-up time suggesting that steroids may only affect the time to recurrence rather than the actual US recurrence rate [8].

Unfortunately, we must consider several limitations when interpretating our study results. Regarding the animal studies, we believe that there is a high chance on publication bias because of current research trends to predominantly publish preclinical studies with positive outcomes [47]. The heterogeneity between studies regarding the type of drug, methods and primary outcomes limits our possibility to draw an unambiguous conclusion. Additionally, creation of US by induction of urethral injury does not fully represent the most common clinical phenotypes with varying aetiologies of US disease. Lastly, the positive outcomes described in almost all preclinical studies cannot be directly extrapolated to a clinical model, as responses to local therapies in an animal model are likely to differ from possible effects in humans.

Limitations of the clinical studies include the fact that many studies reported heterogenous study methods with great differences in type of interventions, application methods and definition of treatment success. This heterogeneity limits the possibility to perform a representable quantitative-analysis as well as increasing the chances of possible bias, mainly due to important differences in the definition used for US recurrence. Also, trials assessing CIC applied different treatment regimens with varying duration and frequency of catheterization which makes it difficult to distinguish the true chemical effects from the mechanical effects of CIC. Furthermore, follow-up time in human studies was generally set at 12–24 months with no data available on yearlong effects of additional treatments. Table 2 shows that MMC and predominantly steroid success rate decrease with increasing follow-up time. As stated in current guidelines, DVIU carries disappointing long-term results which may only temporarily improve with our studied adjuvant therapies [4].

For future research, we recommend a large multicentre RCT to assess the true value of implementation of MMC or HA/CMC in current treatment standards and ensure its clinical safety. Here, method and timing of application should be investigated. Also, the criteria used to define US recurrence have to objective and independent of patients’ preference. Furthermore, it could be useful to explore clinical efficacy of other low cost therapies such as steroids on US with different aetiologies as these outcomes varied largely throughout included clinical studies. Possibly, outcomes for steroid application improve with inclusion of patients with specific stricture aetiologies. Finally, some of the interventions in preclinical studies, such as dexpanthenol and cell-derived materials, may be promising for clinical purposes. Although it could be considered to further assess clinical efficacy, inclusion of a cost effectivity analysis should be added as cell derived materials come with higher costs.

In conclusion, this systematic review shows that local adjuvant therapeutics may have a positive effect on current unsatisfying US recurrence rates after endoscopic procedures. Although MMC and HA/CMC seem to carry the highest clinical potential, clinical effects may decrease over time which underlines the need for a large, well-designed RCT with a yearlong follow-up before considering clinical implementation.

## Supporting information

S1 Checklist PRISMA 2009 checklist. (DOC)

S1 File Search terms. *Literature searches conducted in December 2020 and August 2021*. **S1 Table**: Search terms with number of results per search.
(DOCX)

S2 File In- and exclusion criteria. **S2 Table**: In- and exclusion criteria used during both title/abstract and full-text screen.
(DOCX)

S3 File Outcomes RoB analyses. **S3 Table**: Outcomes RoB analysis using SYRCLE’s tool for animal studies (9).
(DOCX)

S4 File Outcomes RoB analyses. **S4 Table**: Outcomes RoB analysis using RoB-2 tool for randomized controlled trials (10). **S5 Table**: Outcomes RoB analysis using ROBINS-I tool for clinical controlled trials (11).
(DOCX)

## Abbreviations

CIC: clean intermittent catheterization
DVIU: direct visual internal urethrotomy
ECM: extracellular matrix
EPO: erythropoietin
FU: follow-up
HA/CMC: hyaluronic acid and carboxymethylcellulose
MMC: mitomycin-C
RCT: randomized controlled trial
RoB: risk of bias
US: urethral stricture
