# Supplementary material for: The use of local therapy in preventing urethral strictures: A systematic review
Source: PLoS One. 2021 Oct 6;16(10):e0258256. doi: 10.1371/journal.pone.0258256 (PMC8494308; doi:10.1371/journal.pone.0258256)
Supplement: S1 File — Literature searches conducted in December 2020 and August 2021. S1 Table: Search terms with number of results per search. (DOCX) [file pone.0258256.s002.docx]

**Supplementary files and figures**

Supplementary file S1: Search terms

*Literature searches conducted in December 2020 and August 2021.*

| **Database** | **Search terms** | **Number of results** |
| --- | --- | --- |
| Embase* | 'urethral stricture'/de OR 'urethral stricture formation':ab,ti OR 'urethra'/de OR 'urethra' OR 'urothelium'/de AND ('wound healing'/de OR regeneration:ab,ti OR prevention:ab,ti OR 'pharmaceutical preparations') | 1737 |
|  | 'urethral stricture':ab,ti OR 'urethra stenosis'/exp/mj OR 'urethra stenosis'/mj OR 'urethra'/exp OR 'urethra'/de AND ('regeneration'/exp/mj OR 'regeneration'/mj OR 'drug therapy'/exp OR 'drug therapy' OR 'wound healing'/exp OR 'wound healing'/exp/mj OR 'wound healing'/mj) AND ([dutch]/lim OR [english]/lim) AND [abstracts]/lim AND [1987-2017]/py AND ('bladder neck stenosis'/exp OR 'bladder neck stenosis'/de OR 'lower urinary tract symptom'/exp OR 'lower urinary tract symptom'/de OR 'postoperative complication'/exp OR 'postoperative complication'/de OR 'scar formation'/exp OR 'scar formation'/de OR 'side effect'/exp OR 'side effect'/de OR 'urethra stenosis'/exp OR 'urethra stenosis'/de OR 'urine retention'/exp OR 'urine retention'/de) | 1345 |
| Number of results after removal of Embase exact duplicates: 2899 | | |
| PubMed* | ("Urethral Stricture"[Mesh] OR "urethra"[MeSH Terms]) AND ("Wound Healing"[Mesh] OR prevention [tiab]) | 559 |
|  | ("Urethral Stricture"[Mesh] OR "urethra"[MeSH Terms]) AND ("Wound Healing"[Mesh] OR prevention [tiab] OR "stricture formation"[tiab]) | 667 |
|  | ("Urethral Stricture"[Mesh] OR "urethra"[MeSH Terms] OR "urothelium"[Mesh]) AND ("Wound Healing"[Mesh] OR prevention [tiab] OR "stricture formation"[tiab]) | 728 |
|  | ("Urethral Stricture"[Mesh] OR "urethra"[MeSH Terms] OR "urothelium"[Mesh]) AND ("Wound Healing"[Mesh] OR regeneration[tiab] OR prevention [tiab] OR "stricture formation"[tiab]) | 959 |
|  | ("Urethral Stricture"[Mesh] OR "urethra"[MeSH Terms] OR "urothelium"[Mesh]) AND ("Wound Healing"[Mesh] OR regeneration[tiab] OR prevention[tiab] OR "stricture formation"[tiab] OR "Pharmaceutical Preparations"[Mesh]) | 1301 |
|  | ("Urethral Stricture"[Mesh]OR "urethral stricture formation"[tiab] OR "urethra"[MeSH Terms] OR "urothelium"[Mesh]) AND ("Wound Healing"[Mesh] OR regeneration[tiab] OR prevention[tiab] OR "Pharmaceutical Preparations"[Mesh]) | 1197 |
| Number of results after removal of PubMed exact duplicates: 1306 | | |
| Cochrane** | (Urethra):ti,ab,kw OR (urethral stricture):ti,ab,kw OR (urethral stenosis):ti,ab,kw AND ("regeneration"):ti,ab,kw OR (wound healing):ti,ab,kw | 30 |
| Scopus** | ( TITLE-ABS-KEY ( urethral AND stricture ) OR TITLE-ABS-KEY ( urethra ) AND TITLE-ABS-KEY ( wound AND healing ) AND TITLE-ABS-KEY ( local AND treatment ) ) | 26 |
| Web of Science** | urethra (All Fields) or urethral stricture (All Fields) or urethral stenosis (All Fields) AND  (wound healing) OR (regeneration) NOT female SELECTION only articles (no abstracts/book chapters/reviews) | 216 |
| Number of results after removal of Cochrane, Scopus and Web of Science Duplicates: 303 | | |

***** Literature search performed in December 2020
** Literature search performed in August 2021

**Supplementary table 1:** Search terms with number of results per search
