# Supplementary material for: The use of local therapy in preventing urethral strictures: A systematic review
Source: PLoS One. 2021 Oct 6;16(10):e0258256. doi: 10.1371/journal.pone.0258256 (PMC8494308; doi:10.1371/journal.pone.0258256)
Supplement: S2 File — S2 Table: In- and exclusion criteria used during both title/abstract and full-text screen. (DOCX) [file pone.0258256.s003.docx]

Supplementary file S2: In- and exclusion criteria

| **Inclusion criteria** | **Exclusion criteria** |
| --- | --- |
| - English language  - Local therapy  - Outcome includes or is directly related to development/recurrence of urethral strictures  - Experimental or controlled (clinical) trials  - Male subjects | - Language other than English  - Use of stents or scaffolds  - Use of systemic therapy  - Wrong study outcome  - Wrong study design (abstracts, letters, reviews)  - Wrong study population (f.e. inclusion of female subjects)  - No open access  - No control group (human studies) |

**Supplementary table 2:** In- and exclusion criteria used during both title/abstract and full-text screen
