# Supplementary material for: The use of local therapy in preventing urethral strictures: A systematic review
Source: PLoS One. 2021 Oct 6;16(10):e0258256. doi: 10.1371/journal.pone.0258256 (PMC8494308; doi:10.1371/journal.pone.0258256)
Supplement: S3 File — S3 Table: Outcomes RoB analysis using SYRCLE’s tool for animal studies (9). (DOCX) [file pone.0258256.s004.docx]

Supplementary file S3: Outcomes RoB analyses

| **Study** | **Selection bias** | | | **Performance bias** | | **Detection bias** | | **Attrition bias** | **Reporting bias** | **Other** | **Overall** |
| --- | --- | --- | --- | --- | --- | --- | --- | --- | --- | --- | --- |
|  | Sequence generation | Baseline characteristics | Allocation concealment | Random housing | Blinding | Random outcome assessment | Blinding | Incomplete outcome data | Selective outcome reporting | Other sources of bias |  |
| Guzmán *2007* |  |  |  |  |  |  |  | / |  |  |  |
| Krane *2011* |  |  |  |  |  |  |  | / |  |  |  |
| Nagler *2000* |  |  |  |  |  |  |  | / |  |  |  |
| Sangkum *2015* |  |  |  | / |  |  |  | / |  |  |  |
| Shinchi *2019* |  |  |  | / |  |  |  | / |  |  |  |
| Ayyildiz *2004* |  |  |  | / |  |  |  | / |  |  |  |
| Chang *2015* |  |  |  | / |  |  |  |  |  |  |  |
| Fu *2014* |  |  |  | / |  |  |  | / |  |  |  |
| Kurt *2017* |  |  |  | / |  |  |  | / |  |  |  |
| Castiglione *2016* |  |  |  | / |  |  |  |  |  |  |  |
| Nikolavsky *2016* |  |  |  |  |  |  |  | / |  |  |  |
| Sangkum *2016* |  |  |  | / |  |  |  | / |  |  |  |
| Shi *2020* |  |  |  |  |  |  |  |  |  |  |  |
| Chong *2011* |  |  |  | / |  |  |  | / |  |  |  |
| Ayyildiz *2007* |  |  |  |  |  |  |  | / |  |  |  |
| Dündar *2002* |  |  |  |  |  |  |  |  |  |  |  |
| Kilinc *2009* |  |  |  |  |  |  |  | / |  |  |  |
| Sahinkanat *2009* |  |  |  |  |  |  |  | / |  |  |  |
| Yardimci *2015* |  |  |  | / |  |  |  |  |  |  |  |
| Yildizhan *2020* |  |  |  |  |  |  |  |  |  |  |  |

**Supplementary table 3:** Outcomes RoB analysis using SYRCLE’s tool for animal studies (9).
