# Supplementary material for: The use of local therapy in preventing urethral strictures: A systematic review
Source: PLoS One. 2021 Oct 6;16(10):e0258256. doi: 10.1371/journal.pone.0258256 (PMC8494308; doi:10.1371/journal.pone.0258256)
Supplement: S4 File — S4 Table: Outcomes RoB analysis using RoB-2 tool for randomized controlled trials (10). S5 Table: Outcomes RoB analysis using ROBINS-I tool for clinical controlled trials (11). (DOCX) [file pone.0258256.s005.docx]

| **Study** | **Weight** | Randomization process | Deviations from intended interventions | Missing outcome data | Measurement of the outcome | Selection of the reported result | **Overall bias** |
| --- | --- | --- | --- | --- | --- | --- | --- |
| Ergün *2015* | 1 |  |  |  |  |  |  |
| Ali *2015* | 1 |  |  |  |  |  |  |
| Mazdak *2007* | 1 |  |  |  |  |  |  |
| Moradi *2016* | 1 |  |  |  |  |  |  |
| Hosseini *2008* | 1 |  |  |  |  |  |  |
| Mazdak *2010* | 1 |  |  |  |  |  |  |
| Regmi *2018* | 1 |  |  |  |  |  |  |
| Tavakkoli Tabassi *2011* | 1 |  |  |  |  |  |  |
| Chung *2013* | 1 |  |  |  |  |  |  |

**Supplementary table 4:** Outcomes RoB analysis using RoB-2 tool for randomized controlled trials (10).

| **Study** | **Pre-intervention** | | **At intervention** | **Post-intervention** | | | | **Overall bias** |
| --- | --- | --- | --- | --- | --- | --- | --- | --- |
| **Study** | Confounding | Selection of participants into the study | Classification of interventions | Deviations from intended interventions | Missing data | Measurement of the outcome | Selection of the reported result |  |
| Korhonen *1990* |  |  |  |  |  |  |  |  |
| Yildirim *2016* |  |  |  |  |  |  |  |  |
| Shirazi *2007* |  |  |  |  |  |  |  | / |

**Supplementary table 5:** Outcomes RoB analysis using ROBINS-I tool for clinical controlled trials (11).
